# Novel electrospun fibers as carriers for delivering a biocompatible Sm(iii) nanodrug for cancer therapy: fabrication, characterization, cytotoxicity and toxicity†

**DOI:** 10.1039/d2ra06052c

**Published:** 2023-01-11

**Authors:** R. Fouad, Amira A. M. Ali

**Affiliations:** a Department of Chemistry, Faculty of Education, Ain Shams University Roxy Cairo Egypt Raniaahmed@edu.asu.edu.eg +20 02 22581243 +20 01000212207

## Abstract

The current study represents the successful fabrication and characterization of a Sm(iii) nano complex based on 2-cyano-*N*′-((4-oxo-4*H*-chromen-3-yl)methylene)acetohydrazide (CCMA). The fibrous Sm(iii) nanocomplex has been fabricated by the electrospinning technique. SEM analysis of the electrospun fibers has revealed that the fibers have a uniform structure and smooth surface without observing Sm(iii) nanocomplex crystals, *i.e.* the Sm(iii) nanocomplex has been well incorporated into the fibers. *In vitro* antitumor activity against two carcinogenic cell lines (HepG-2 and E.A.C.) as well as *in vivo* toxicity of pure Sm(iii) nanocomplex and its electrospun fibers have been detected. The biological results have shown that there is a significant antitumor activity with low toxicity of the pure Sm(iii) nanocomplex and its electrospun fibers with respect to different standard antitumor drugs. Also, the electrospun fibers recorded higher cytotoxicity (IC_50_ = 0.1 μM (Hep-G); 0.09 μM (E.A.C)) and lower toxicity (LD_50_ = 350 mg kg^−1^) than the pure ones. The *in vitro* release rate of the Sm(iii) nanocomplex from electrospun fibers has also been detected. The results have shown that the burst releasing of the Sm(iii) nanocomplex is about 22% after 1 h at the beginning, then a cumulative release increased gradually over the following hours. All results demonstrate the potential use of the Sm(iii) nanocomplex as a potent antitumor drug and its electrospun fibers as superior drug carriers for the treatment of tumors.

## Abstract 2

The current study represents the successful fabrication and characterization of a Sm(iii) nano complex based on 2-cyano-*N*′-((4-oxo-4*H*-chromen-3-yl)methylene)acetohydrazide (CCMA).

## Graphical abstract

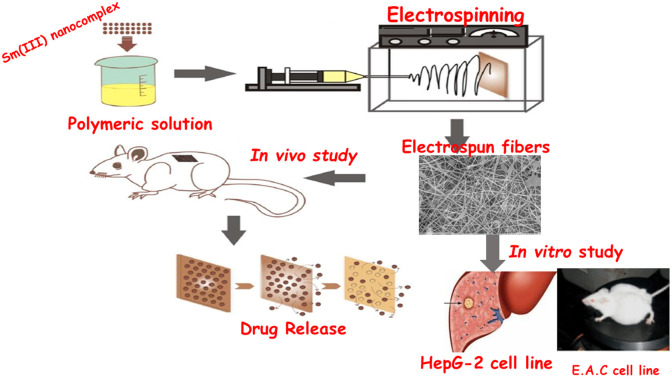

## 1. Introduction

Chemotherapy is the most widely recognized system of cancer therapy. Despite the significant advances in chemotherapy, directly killing tumor cells, it still causes many side effects. This includes mouth sores, fatigue, nausea, immunosuppression, and hair loss. It is, moreover, toxic to immune and healthy living cells.^1–3^ This is the result of the random distribution of chemotherapeutic drugs in healthy cells; as just a small portion of the medication is delivered to tumors.^1^ Thus, the targeted therapy needs substantial improvements such as minimizing the doses to the least amount possible as well as trying to control the side effects of these drugs, while retaining the significant effect of the dose and controlling the dose that penetrates to cells.^4^ In addition, the creation of novel antitumor drugs and various drug-delivery systems have been required to enhance the site specificity and bio-availability of medications, hence, increasing the therapeutic efficacy and reducing systemic toxicity.^5^ With the aim of improving the effectiveness of chemotherapy and decreasing its side effects, the electrospinning technique has been employed for the fabrication of polymer fibers of diameters ranging from a few nanometers to several micrometers with a broad range of applications.^6–8^ This includes nano-drug delivery systems for the treatment of cancer.^9–11^ Nanofibers, loaded as anticancer drugs, can enhance the treatment of cancer that needs a small portion of drugs. This is because drugs are stored in nanofibers for a longer period of time. Moreover, the electrospun nanofibers have several advantages such as large surface area, controllable pore size, small diameter, and high aspect ratio. They are also characterized by their biodegradability, flexibility, and are suitable bioavailability carriers. Electrospun nanofibers possess high compatibility for the encapsulation of drugs and the possibility of modulating drug release.^12–17^ Therefore, the fabrication of nanofiber drugs by electrospinning can be considered a new strategy to produce antitumor, drug-delivery nanomaterials.

Lanthanide (Ln(iii)) complexes have attracted the attention of medicinal inorganic chemists due to their several biological uses including antibacterial, antifungal, antiviral, and antitumor activities.^18–21^ The cytotoxicity of lanthanide complexes is typically due to interactions between lanthanides and DNA, blockage of calcium transport in mitochondria, and endoplasmic reticulum stress pathway-mediated apoptosis. Inhibiting the reductase thioredoxin and targeting the pathway of the glutathione-independent lipoate reduction using gadolinium(iii) texaphyrin (MGd, Xcytrin) is the other important mechanism in which, subsequently inhibits the replication of cancer cells in DNA, repairs and induces oxidative stress. Lanthanide nanomaterials are not only small-sized molecules but had also *in vitro* cytotoxic effects against different human cancer cell lines such as pancreatic carcinoma, hepatocellular and squamous cell carcinoma. This toxicity may be due to oxidative stress, activating the mitogen-activated protein kinase (MAPK), as well imitations of superoxide dismutase, glutathione peroxidase, and catalase activity studies.^22,23^ Moreover, functional nanofibers containing lanthanide complexes will effectively improve the biological activity of pure metal complexes.^1^ Till now, the majority of reports about Sm(iii) complexes and their biomedical applications are in the bulk state.^24–29^ The reports about the incorporation of Sm(iii) complexes into polymeric nanofibers are, however, limited. Therefore, an essential goal of the present study is the fabrication of a drug delivery system by preparation of electrospinning fibers (as a biocompatible drug carrier) with loading Sm(iii) nanocomplex, their antitumor activities were evaluated both *in vitro* on tumor cell lines and *in vivo* on tumor-bearing mice.

## 2. Experimental

### 2.1. Material and techniques

In this work, all chemicals and solvents used are of analytical reagent grade (AR). Anhydrous Samarium nitrate and sodium acetate have been obtained from Sigma-Aldrich. Polyvinyl alcohol (PVA, MW = 1500 g mol^−1^) has been obtained from Sigma-Aldrich. Organic solvents have been spectroscopic pure from BDH including absolute ethanol, diethyl ether, dimethyl formamide (DMF) and phosphate-buffered saline (PBS, pH 7.4).

Elemental analysis (C, H, N percentage) has been operated by Vario El-Elementar (Cairo University, central laboratory). Melting and decomposition temperatures of the ligand and its complex have been detected by the Stuart melting point. Corning conductivity meter NY 14831 model 441 has been used to detect a molar conductance for 10^−3^ M (DMF) of Sm(iii) nanocomplex. Using KBr discs, a Nicolet 6700 FT-IR spectrometer has been applied to record the FT-IR spectrum of Sm(iii) nanocomplex. The luminescence spectral data of the prepared materials have been detected on a PerkinElmer LS 55 luminescence spectrometer (USA). Thermal gravimetric analysis (TG) has been measured on the Shimadzu-50 instrument. TG data has been recorded from ambient temperature up to 800 °C under N_2_ flow at a heating rate of 10 °C min^−1^. Sm(iii) nanocomplex has also been characterized by transmission electron microscope (TEM, JEM-2100(JEOL)), operated at 200 kV accelerating voltage. QUANTA FEG 250 instrument of electron microscopy (SEM) has been used to characterize the electrospun fibers. UV-Vis spectrophotometer (a JASCO model V-550) has been used for recording electronic spectra of Sm(iii) nanocomplex.

### 2.2. Synthesis

#### 2.2.1. Synthesis of 2-cyano-*N*′-((4-oxo-4*H*-chromen-3-yl)methylene)acetohydrazide (CCMA)

The organic ligand (CCMA) has been successfully prepared according to the literature method.^30^

Anal. found (calcd) for C_13_H_9_N_3_O_3_: M.wt: (255); % C: 61.20 (61.17); % H: 3.54 (3.55); % N: 16.45 (16.47); IR (KBr cm^−1^): *ν*(C=O): 1692, *ν*(C=O)_γ-pyrane_: 1633; *ν*(CH=N): 1564; *λ* (nm): 268, 304, 423.

#### 2.2.2. Synthesis of pure Sm(iii) nanocomplex

**Scheme 1:**
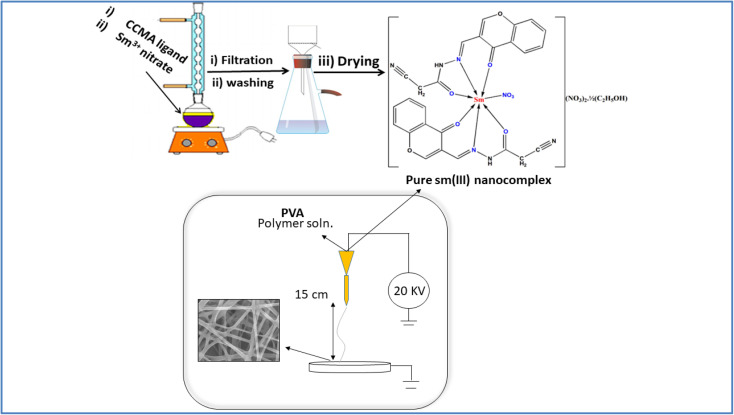
Schematic representation of Sm(iii) nanocomplex and electrospun fibers.

The pure Sm(iii) nanocomplex has been prepared by adding an aqueous solution of anhydrous metal nitrate salt to a hot ethanolic solution of CCMA ligand in presence of sodium acetate as a buffering agent. The mixture has been refluxed for 8 h. The separated Sm(iii) nanocomplex has, then, been filtered and washed with ethanol and ether and dried in vacuum desiccators over anhydrous CaCl_2_ (Scheme 1).

The obtained complex is [Sm(CCMA)_2_(NO_3_)](NO_3_)_2_·1/2(C_2_H_5_OH); yield: 85%; M.P (°C): >300; anal. found (calcd) for C_27_H_21_N_9_O_15.5_Sm: M.wt: (869); % C: 37.18 (37.28); % H: 2.33 (2.43); % N: 14.51 (14.49); IR (KBr cm^−1^): *ν*(OH)_(ethanolic)_: 3385_br_; *ν*(C=O): 1645, *ν*(C=O)_γ-pyrane_: 1612; *ν*(CH=N): 1545; *ν*(M–O): 604_m_, 590_m_, 545_m_; *ν*(M–N): 484, 424; *ν*(NO_3_)_coord_: 1300, 1106; *ν*(NO_3_)_ionic_: 1384, 867; molar conductance (*Λ*_m_): 99 ohm^−1^ mol^−1^ cm^2^; *λ* (nm): 260, 302, 400.

#### 2.2.3. Synthesis of electrospun fibers of Sm(iii) nanocomplex

Electrospun nanofibers of Sm(iii) nanocomplex has been prepared according to the prescribed methodology.^31^ First, PVA solution (14 wt%) has been prepared by dissolving PVA gradually in distilled water at 80 °C with vigorous stirring for 1 h. Then, (1 wt%) of Sm(iii) nanocomplex has been added to the previous solution with continuous stirring at 80 °C. The resultant colloidal mixture has, then, been stirred for 3 h. A high-voltage power supply has been operated as the source of the electric field. The colloidal solution has been introduced through a plastic syringe attached to a capillary needle; a positive electrode (anode) connected with the colloidal solution through the capillary needle, and a negative electrode (cathode) was attached to a metallic collector which has been covered with an aluminum sheet. The resultant solution has been typically electrospun at 20 kV and 15 cm working distance (the distance between the needle tip and the metallic collector). The formed fiber sample has, finally, been dried in a vacuum desiccator over anhydrous CaCl_2_ (Scheme 1).

### 2.3. Biological study

#### 2.3.1. *In vitro* drug release

A vial has been filled with a piece of Sm(iii) nanocomplex-loaded fiber (30 mg) and 100 mL phosphate-buffered saline (PBS, pH 7.4). A thermostat shaker has been used to incubate the vial at 37 °C. After incubation, 1.0 mL of the released solution has been extracted from the dissolving medium and an equal amount of fresh PBS has, then, been added back to the incubation solution. The concentration of Sm(iii) nanocomplex in the release media has been, finally, detected by an ultraviolet spectrophotometer.

#### 2.3.2. Antitumor activity (*in vitro* study)

The *in vitro* antitumor activities of Sm(iii) nanocomplex (pure) and its electrospun fibers have been tested against two cell lines: human hepatocellular carcinoma (HepG-2) and Erlish cities carcinoma (E.A.C). The detection of cytotoxic activity has been based on the change in cell viability and the distortion of the morphology of cells according to the literature technique.^32,33^

#### 2.3.3. Acute toxicity (*in vivo* study)

The toxicity of pure and electrospun fibers of Sm(iii) nanocomplex were performed at the Regional Center for Mycology and Biotechnology (RCMB), Al-Azhar University, Cairo, Egypt. Mice have been kept in groups of ten in plastic airy cages with unrestricted access to food and water under controlled environmental conditions (12 h of darkness and 12 h of light at 25 °C). Subsequently, five dosages have been selected to determine the injectable muscles LD_50_ in mice. They have, then, been administered to five groups of albino mice (10 in each group). The number of dead mice in each group has been counted after 24 hours. To calculate the LD_50_ (median lethal dose), which is the dosage or concentration that results in the death of 50% of the experimental animals, the proportion of animals that died at each dose level has been converted.^34^

## 3. Result and discussion

### 3.1. Structure, thermal, mass descriptions

A pure Sm(iii) nanocomplex has been, successfully, prepared in a good yield with the empirical formula: [Sm(CCAM)_2_(NO_3_)](NO_3_)_2_·1/2(C_2_H_5_OH). The pure complex has been stable in the air with a yellow crystalline form. It is sparingly soluble in methanol and ethanol but soluble in DMF and DMSO solvents. The pure complex has been decomposed up to 300 °C. According to analytical data, the chemical structure of pure Sm(iii) nanocomplex has been proposed as the stoichiometric ratio of metal : CCMA is 1 : 2 (Scheme 1). The molar electric conductivity has been in the range of 1 : 2 electrolyte which confirms the presence of two ionic nitrates out of the coordination sphere as counter ions.^35^ The mass spectrum has been used to scrutinize the proposed stoichiometry (Fig. S1†). Pure Sm(iii) nanocomplex exhibits a molecular ion peak at *m*/*z* 869 which agrees with the theoretical formula. Also, there is a good confirmation between analytical, mass spectral data suggested theoretical formula and thermal data. The TG curve of pure Sm(iii) nanocomplex is depicted in Fig. S2.† The half ethanolic molecule has been released at 92 °C with weight loss of found; (calcd) 2.65% (2.64%). The three molecules of NO_2_ have, then, been released at elevated temperature (342 °C) with weight loss of found; (calcd) 18.55% (18.51%).

### 3.2. Spectral description

FT-IR spectral data of pure Sm(iii) nanocomplex has shown the characteristic spectral bands of (C=O)_carbonyl_, (C=O)_γ-pyrane_ and (CH=N) groups which are shifted from CCMA ligand spectral bands, indicating the participation of these groups in the complexation. Also, the participation of (C=O)_carbonyl_ and (C=O)_γ-pyrane_ have been also confirmed by the appearance of new bands at 609, 590 and 545 cm^−1^ which is assigned to *ν*(M–O) bond.^36,37^ In addition, the coordination of the nitrogen atom of the azomethine group to the central metal atom has also been indicated by the presence of new bands at 424 and 484 cm^−1^ due to *ν*(M–N).^38–40^ The presence of coordinated nitrate group is evidenced by the appearance of new bands at 1300 and 1106 cm^−1^ due to asymmetric and symmetric *ν*_1_, *ν*_2_ vibration modes, respectively.^37,41^ The ionic nitrate groups have been confirmed by the appearance of new bands at 1384 and 867 cm^−1^.^41^ This observation agrees with the molar conductance data.

According to previous FT-IR results, CCMA ligand behaves as neutral tridentate through O atoms of the carbonyl group and carbonyl of γ-pyrane, N atom of CH=N group.

The electronic absorption spectral data of CCMA has shown the absorption bands at 268 and 304 nm. The highest energy bands have been assigned to π–π* transitions within aromatic rings.^42,43^ Moderate energy bands can be assigned to n–π* transitions within C=O and C=N groups.^44,45^ The CT band has also been observed at 423 nm. Upon complexation, the blue shift has been recorded for the electronic absorption bands. The electronic absorption spectrum of pure Sm(iii) nanocomplex has shown bands at 260, 302 and 400 nm. Also, the electrospun fibers of Sm(iii) nanocomplex have shown bands at 245, 273 and 352 which obey the blue shift with respect to the pure one.

### 3.3. Morphological description

**Fig. 1:**
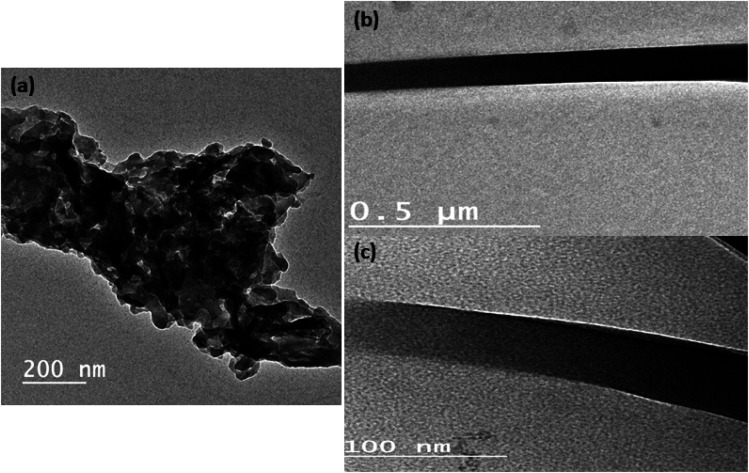
TEM image of (a) Sm(iii) nanocomplex; (b) and (c) TEM image of electrospun fibers with high and low magnification, respectively.

**Fig. 2:**
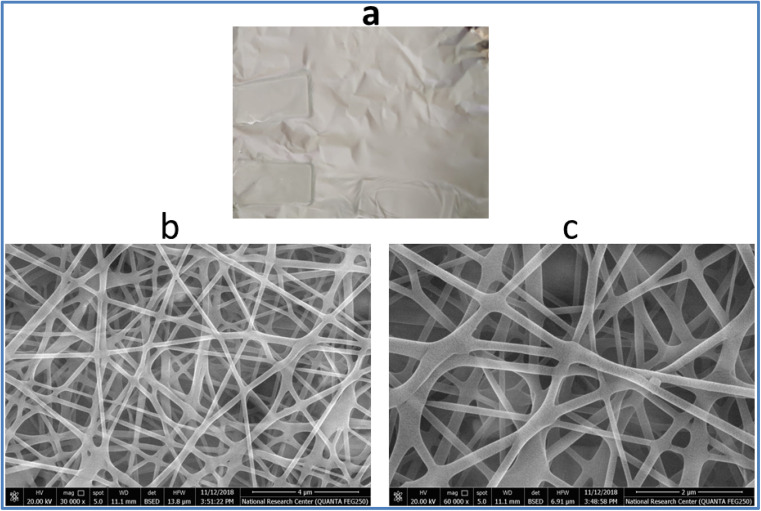
(a) The photograph of macromorphology of electrospun fibers of Sm(iii) nanocomplex; (b) and (c) SEM image of electrospun fibers of Sm(iii) nanocomplex with low and high magnification, respectively.

Fig. 1a and b shows the TEM image of pure Sm(III) complex and Sm(iii) complex nanofiber. Sm(iii) complex has non-uniform aggregated nanosheet morphology (28 nm) (Fig. 1a). The TEM image of Sm(iii) complex nanofiber cleared hybrid micro and nano rode morphology (Fig. 1b). The micromorphology of Sm(iii) complex nanofiber membrane looks like white paper in natural light (Fig. 2a). Electrospun fibers SEM images of Sm(iii) nanocomplex were collected in Fig. 2b and c. The images have shown that the fibers have a uniform, straight, flexible and smooth structures without beads owing to the good electrospinning conditions. The smooth surface has indicated that the Sm(iii) complex is well incorporated in polymeric fibers. The fibers have shown different diameter ranges from nano to micro scale (95 to 148 nm)_average_. Also, the fibers have been aligned in random orientation, which are caused by the bending instability associated with the spinning jet. The fibers have linked voids that together form a 3D fibrous network.

### 3.4. Biological study

#### 3.4.1. *In vitro* drug delivery

**Fig. 3:**
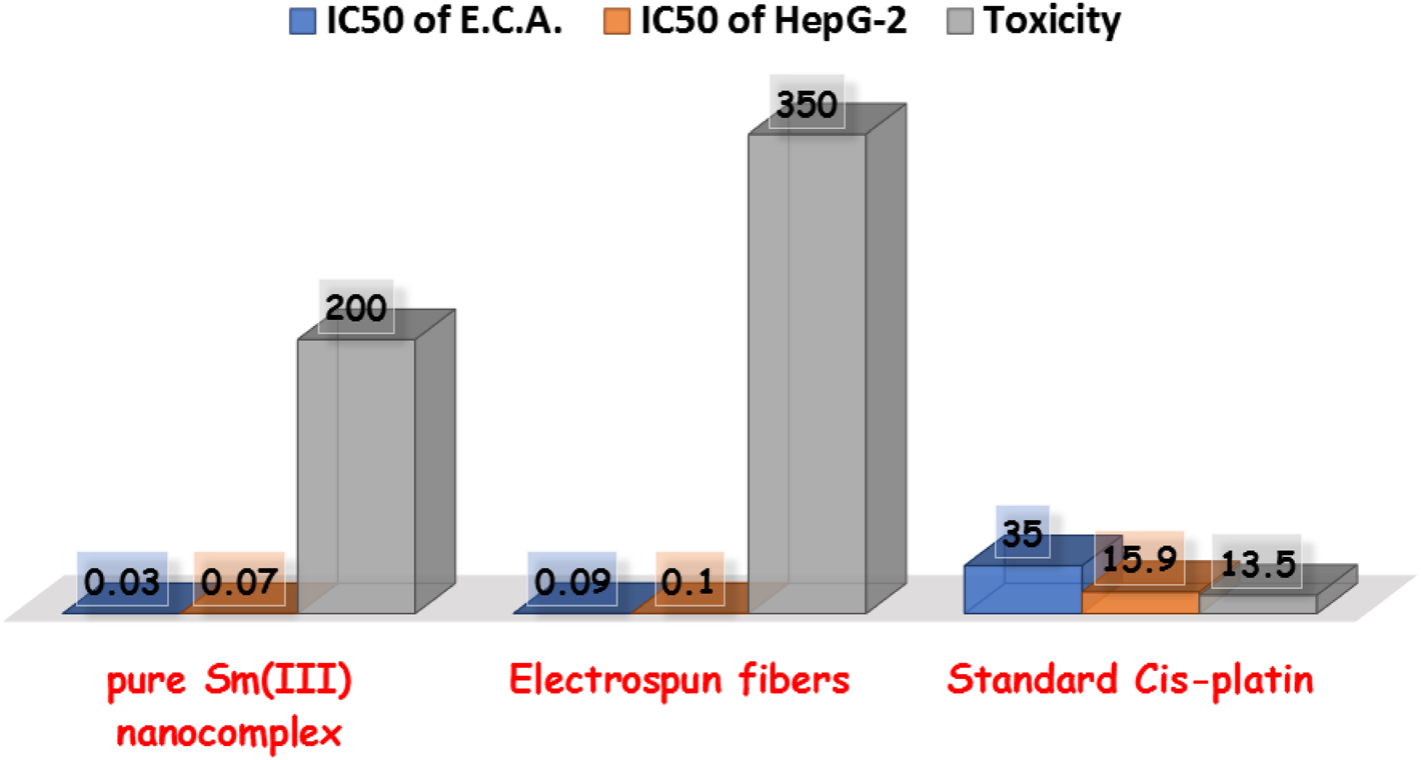
*In vitro* cytotoxicity of pure Sm(iii) nanocomplex, its electrospun fibers, and standard cis-platin on Ehrlich ascites carcinoma cell line (blue columns); on HepG-2 carcinoma cell line (orange columns); toxicity of pure Sm(iii) nanocomplex and its electrospun fibers on mice in comparison with cis-platin (gray columns).

The *in vitro* rate release of Sm(iii) nanodrug from electrospun fibers has been followed by spectrophotometry. The fiber has been immersed in phosphate-buffered saline (PBS) solution with pH of 7.4, at a controlled temperature of 37 °C and the absorbance of the prepared solution has been measured by spectrophotometer as a function of time. Fig. 3 shows the profile release of Sm(iii) nanodrug from electrospun fibers. As time increase, the absorption peak intensity increases due to increasing the drug release process according to a diffusion-controlled mechanism.^46^ Sm(iii) nanocomplex released from the electrospun fibers showed a burst release (about 22%) during the first 1 h, releasing a considerable amount of drug during the first 60 min. Afterward, a gradual increase in the cumulative release followed, over the next hours, reaching a plateau at 30 h (Fig. 3). This burst release may be due to the presence of Sm(iii) nanocomplex on or near the surface of the electrospun fibers. The considerable amount of Sm(iii) nanocomplex could be easily diffused, causing the burst effect. In conclusion, electrospun fibers really demonstrate a rapid and a significant release at the very beginning and then provide a continuous release of Sm(iii) nanocomplex based on the diffusion mechanism. These results strongly suggest that the electrospun fibers have an effect of controlled release of Sm(iii) nanodrug and are effective for cancer therapy.

### 3.5. Cytotoxic activity

**Fig. 4:**
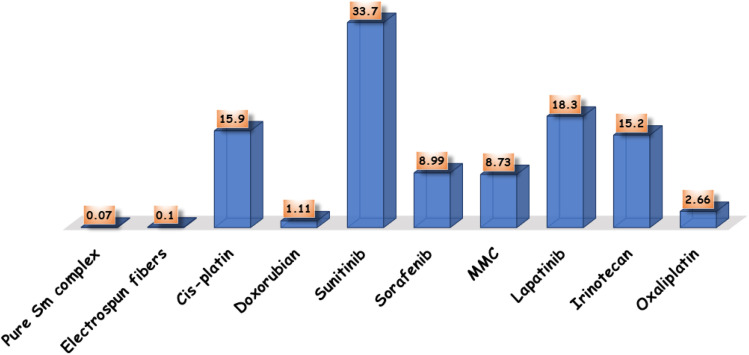
Comparison between IC_50_ of pure Sm(iii) nanocomplex, its electrospun fibers and standard antitumor drugs.

Cell lines have been evaluated to detect the cytotoxic activities of pure Sm(iii) nanocomplex and its electrospun fibers against HepG-2 and E.A.C. To detect the cytotoxic efficiency of pure Sm(iii) nanocomplex and its electrospun fibers, IC_50_ values should be calculated. Fig. 4 summarizes the cell viability against inhibitory concentration (IC_50_) to show the effectiveness of the prepared compound to inhibit the growth of tumor cells. As observed in Fig. 3, pure Sm(iii) nanocomplex and its electrospun fiber can be used to treat cell line carcinoma at concentrations of 0.07 μM (HepG-2); 0.03 μM (E.A.C) and 0.10 μM (HepG-2); 0.09 μM (E.A.C), respectively. The blank fiber carrier PVA without Sm(iii) nanocomplex has not recorded any cytotoxicity. The results have also shown that the newly prepared compounds exhibit strong inhibition activity on tested cell lines with respect to standard antitumor drugs and literature-prepared antitumor compounds.^24,47–49^

**Fig. 5:**
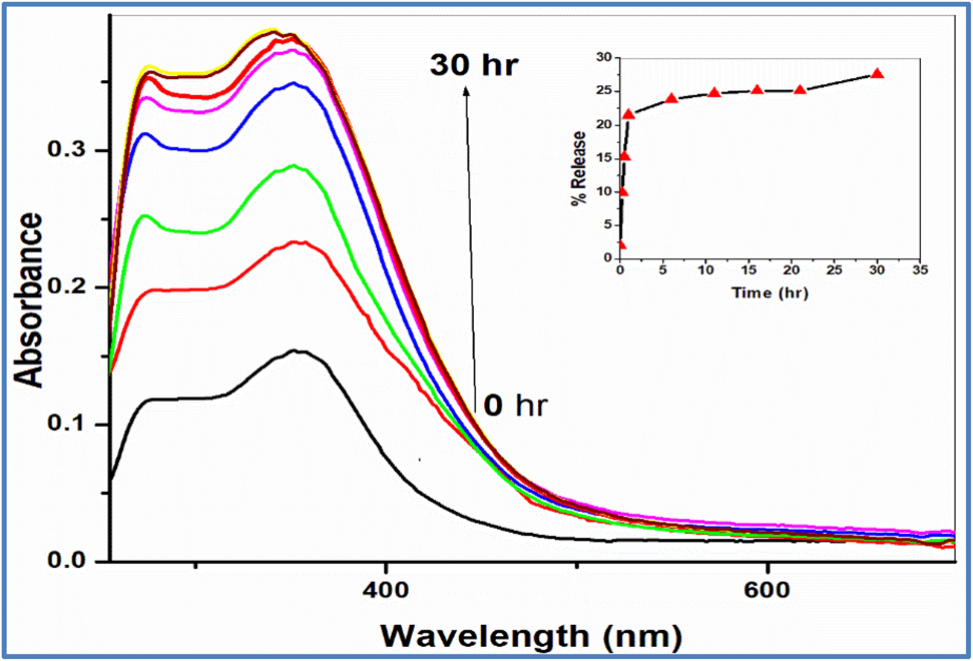
The profile release of Sm(iii) nanodrug from electrospun fibers; inset: the relation between % release of Sm(iii) nanodrug and time.

At first sight to cytotoxic values, the pure Sm(iii) nanocomplex has higher activity than its electrospun fibers. However, a deeper look at these values shows that the electrospun fibers of Sm(iii) nanocomplex is more active than pure ones. The significantly highest activity of electrospun fibers is attributed to the low concentration of Sm(iii) nanocomplex which is incorporated into the PVA carrier (see Experimental section). So, when holding the comparison of the cytotoxic activity of the compounds with respect to the concentration of Sm(iii) nanocomplex, it is deduced that the highest cytotoxic effect is that of electrospun fibers. Also, the concentration of Sm(iii) nanocomplex, at which IC_50_ has been detected, refers to the concentration of pure Sm(iii) nanocomplex only. But in the case of electrospun fibers, PVA solution (14 wt%) has been prepared, then, (1 wt%) of Sm(iii) nanocomplex has been added to the previous solution. Fig. 5 represents a good comparison between pure Sm(iii) nanocomplex and its electrospun fibers and commercial antitumor drugs against HepG-2 cell lines. It is clear that pure Sm(iii) nanocomplex and its electrospun fibers have higher activity than cis-platin (IC_50_ = 15.9 μM), doxorubian (IC_50_ = 1.1 μM), sunitinib (IC_50_ = 33.7 μM), sorafenib (IC_50_ = 8.99 μM), MMC (IC_50_ = 8.73 μM), lapatinib (IC_50_ = 18.3 μM) and oxaliplatin (IC_50_ = 2.66 μM).^36,50^ Also, the electrospun fibers have recorded the highest cytotoxic activity with respect to its pure one and diverse commercial antitumor drugs. The highest antitumor of electrospun fibers may be assigned to its morphology (uniform size and smoothing surface) as shown in Fig. 2b, which facilitates its penetration into tumor cells and damages them. Consequently, the electrospun fibers have potential inhibitory efficiency for therapeutic tumors and could be considered a promising candidate as drug carriers for local treatment of tumors.

### 3.6. Acute toxicity

The toxicity of pure and electrospun fibers of Sm(iii) nanocomplex has been detected on mice. The Lethal dose (LD_50_) for pure Sm(iii) nanocomplex and its fibers have been calculated as 200 and 350 mg per kg body weight, respectively (Fig. 4). These results have indicated that pure Sm(iii) nanocomplex and its fiber are less toxic with respect to standard cis-platin (LD_50_ = 13.5 mg kg^−1^).^51,52^ The lowest toxicity with the highest cytotoxicity of electrospun Sm(iii) fiber makes it a promising biocompatible antitumor agent impeded in polymeric delivery host to help in using it as a biocompatible agent for drug delivery.

## 4. Conclusion

The present study improves both safety and efficacy of cancer chemotherapy and overcomes its shortcomings. Pure Sm(iii) nanocomplex has been prepared by refluxing Sm(NO_3_)_3_ with CCMA ligand. Also, its electrospun fibers have been prepared by using the electrospinning technique to control the release of anticancer drugs (Sm(iii) nanocomplex). The electrospun fibers have seemed uniform with the smoothing surface and no Sm(iii) nanocomplex crystals have been observed. Sm(iii) nanocomplex has been finely incorporated in the fibers. The *in vitro* antitumor activity has been measured against two carcinogenic cell lines (HepG-2 and E.A.C.) and the *in vivo* toxicity of pure Sm(iii) nanocomplex and its electrospun fibers have been detected. The results show that the highest antitumor activity with the lowest toxicity of Sm(iii) nanocomplex incorporated in fibers with respect to its pure one and standard antitumor drugs. From the *in vitro* rate release of Sm(iii) nanodrug from electrospun fibers, it is shown that a burst releasing of the drug has been observed during the first hour (about 22%). Afterward, the cumulative release increased gradually over the next hours, reaching a plateau at 30 h. These results strongly suggest that the electrospun fibers have an effect of controlled release of Sm(iii) nanodrug and are effective for cancer therapy.

## Ethical statement

Animal dealing conditions and treatment were guided as per the National Institute of Health Guide for Animal and approved by the Institutional Animal Care and Use Committee (IACUC).^53^

## Conflicts of interest

The authors declare that they have no known competing financial interests or personal relationships that could have appeared to influence the work reported in this paper.

## Supplementary Material

RA-013-D2RA06052C-s001
