# Supplementary figures and images for: Novel electrospun fibers as carriers for delivering a biocompatible Sm(iii) nanodrug for cancer therapy: fabrication, characterization, cytotoxicity and toxicity
Source: RSC Adv. 2023 Jan 11;13(3):1883–91. doi: 10.1039/d2ra06052c (PMC9832981; doi:10.1039/d2ra06052c)

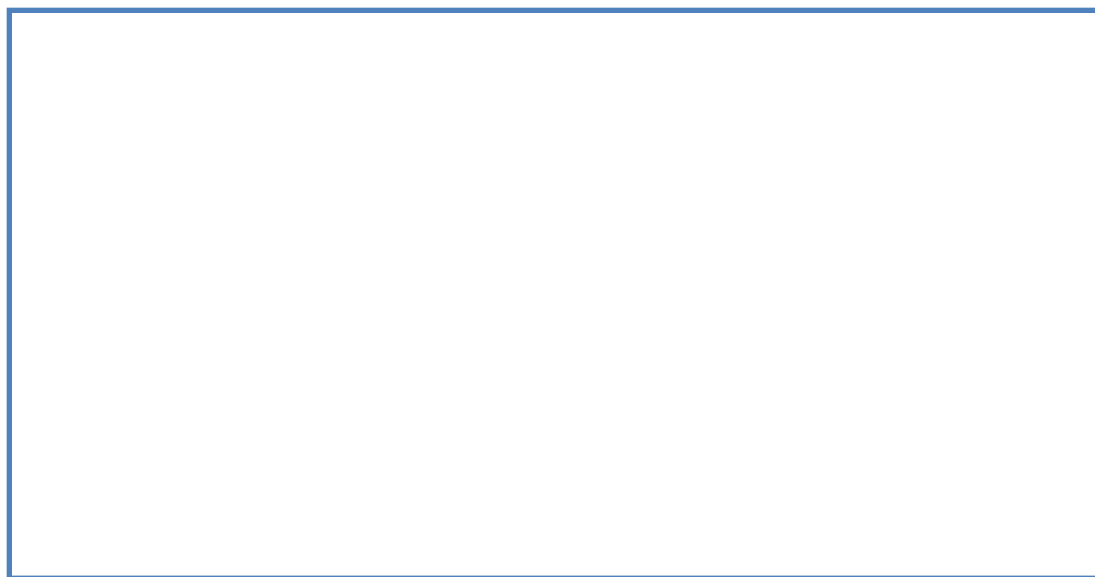

**Fig. S1.** Mass spectrum of Sm(III) nanocomplex

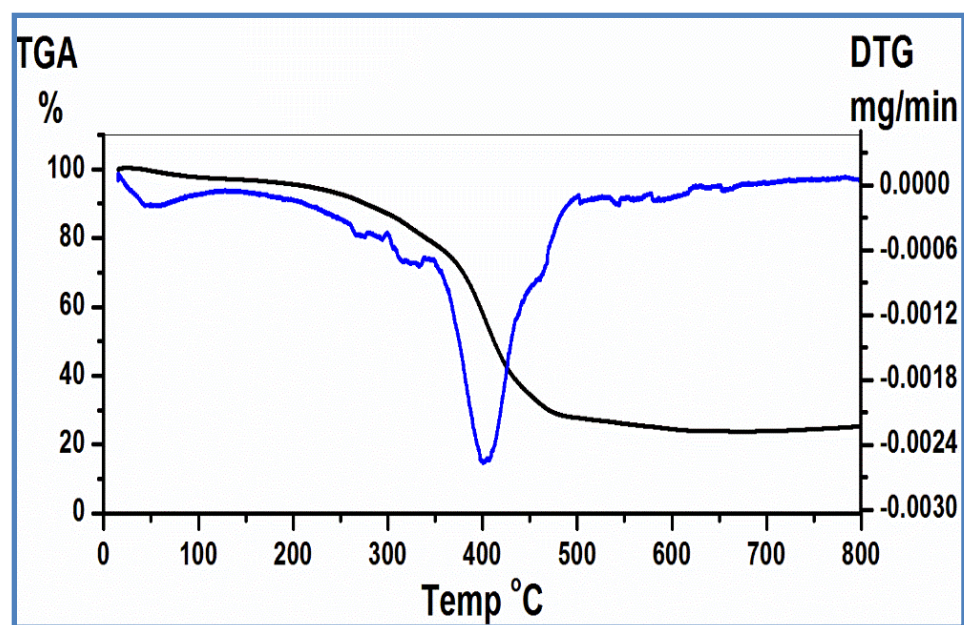

**Fig. S2.** TGA curve of Sm (III) nanocomplex

Supplement: RA-013-D2RA06052C-s001 [file RA-013-D2RA06052C-s001.pdf]
